# Microstructural Integrity of the Superior Cerebellar Peduncle Is Associated with an Impaired Proprioceptive Weighting Capacity in Individuals with Non-Specific Low Back Pain

**DOI:** 10.1371/journal.pone.0100666

**Published:** 2014-06-20

**Authors:** Madelon Pijnenburg, Karen Caeyenberghs, Lotte Janssens, Nina Goossens, Stephan P. Swinnen, Stefan Sunaert, Simon Brumagne

**Affiliations:** 1 KU Leuven Department of Rehabilitation Sciences, University of Leuven, Leuven, Belgium; 2 Department of Physical Therapy and Motor Rehabilitation, University of Ghent, Ghent, Belgium; 3 Department of Movement and Sports Sciences, University of Ghent, Ghent, Belgium; 4 KU Leuven Department of Kinesiology, University of Leuven, Leuven, Belgium; 5 KU Leuven Department of Imaging and Pathology, University of Leuven, Leuven, Belgium; The University of Queensland, Australia

## Abstract

**Introduction:**

Postural control is a complex sensorimotor task that requires an intact network of white matter connections. The ability to weight proprioceptive signals is crucial for postural control. However, research into central processing of proprioceptive signals for postural control is lacking. This is specifically of interest in individuals with non-specific low back pain (NSLBP), because impairments in postural control have been observed as possible underlying mechanisms of NSLBP. Therefore, the objective was to investigate potential differences in sensorimotor white matter microstructure between individuals with NSLBP and healthy controls, and to determine whether the alterations in individuals with NSLBP are associated with the capacity to weight proprioceptive signals for postural control.

**Methods:**

The contribution of proprioceptive signals from the ankle and back muscles to postural control was evaluated by local muscle vibration in 18 individuals with NSLBP and 18 healthy controls. Center of pressure displacement in response to muscle vibration was determined during upright standing on a stable and unstable support surface. Diffusion magnetic resonance imaging was applied to examine whether this proprioceptive contribution was associated with sensorimotor white matter microstructure.

**Results:**

Individuals with NSLBP showed a trend towards a reduced fractional anisotropy along the left superior cerebellar peduncle compared to healthy controls (p = 0.039). The impaired microstructural integrity of the superior cerebellar peduncle in individuals with NSLBP was significantly correlated with the response to ankle muscle vibration (p<0.003).

**Conclusions:**

In individuals with NSLBP, a decreased integrity of the superior cerebellar peduncle was associated with an increased reliance on ankle muscle proprioception, even on unstable support surface, which implies an impaired proprioceptive weighting capacity. Our findings emphasize the importance of the superior cerebellar peduncle in proprioceptive weighting for postural control in individuals with NSLBP.

## Introduction

Low back pain is a major and worldwide health problem [1], [2]. Approximately 85% of the low back pain complaints have a non-specific character, which implies that no specific cause or underlying mechanism can be identified [1], [3]. As a result, the effects of current low back pain interventions are rather small [2]. Therefore, research into the mechanisms and causes of non-specific low back pain (NSLBP) was recently considered as one of the priorities in low back pain research [4].

Impairments in postural control, the ability to control the upright body posture in a variety of environmental conditions by using sensory input from the visual, vestibular and proprioceptive system [5], [6], have been studied as possible factors that cause and maintain NSLBP [7]. Many studies in the field of postural control and NSLBP have focused on the motor output [7]. However, sensory input, more specifically the ability to weight proprioceptive signals adaptively, is considered to be crucial for postural control [8]–[10].

Proprioception can be defined as ‘the unconscious perception of movement and spatial orientation arising from stimuli within the body’ [11]. Several studies showed a compromised back muscle proprioception in individuals with NSLBP [12]–[15]. Research in this field has mainly concentrated on the peripheral aspects of proprioception, i.e. muscle spindles. Specifically, peripheral stimulation of muscle spindles has revealed a consistent increase of the gain of ankle muscle proprioception over back muscle proprioception in individuals with NSLBP, which indicates an impaired proprioceptive weighting capacity [16], [17]. In challenging conditions such as standing on unstable support surfaces, ankle muscle proprioception becomes less reliable [18]. Consequently, an efficient weighting capacity of sensory signals down-weights the proprioceptive signals from the ankles and up-weights the proprioceptive signals from more proximal segments, such as the back region, to provide an optimal postural control [18]. In individuals with NSLBP, sensory integration is less optimal due to the deterioration of proprioceptive input from the back muscles, which results in impaired postural control [16], [17], [19], [20]. This proprioceptive deterioration and subsequent impaired postural control might contribute to spinal loading, pain and recurrence of NSLBP [21].

Although considerable research has been devoted to the sensory input at peripheral level, rather less attention has been paid to the processing of these sensory signals at central level. However, postural control is a complex sensorimotor task that requires an intact network of white matter connections [22]. The white matter microstructure can be revealed by diffusion magnetic resonance imaging (diffusion MRI). Buckalew et al. showed that alterations in white matter microstructure were related to disability in older individuals with NSLBP [23]. In addition, several studies focused on the association between white matter integrity and pain chronification in NSLBP [24], [25]. However, to the author’s best knowledge, attempts to establish the link between white matter microstructure and sensorimotor control in individuals with NSLBP are lacking. In other populations, such as patients with traumatic brain injury [26] and multiple sclerosis [22], a relationship between their reduced integrity of white matter pathways and impaired postural control has been found. However, no differentiation between the sensory and motor level of postural control was possible in these studies. In other words, it remains unclear whether changes in the integrity of sensorimotor white matter pathways are related to the proprioceptive weighting capacity for postural control, and more specifically in the NSLBP population.

On the other hand, research on gray matter characteristics emphasizes that central sensorimotor changes are present in individuals with NSLBP. Specifically, individuals with chronic low back pain have an increased cortical thickness of the primary somatosensory cortex [27]. Moreover, the primary somatosensory cortex shows an altered somatotopic organization based on tactile input. More specifically, the sensory localization of the back region has shifted into the medial and inferior direction, indicating an expansion towards the cortical representation of the leg [28]. Likewise, a reorganization of the motor cortex is observed in the NSLBP population and is associated with postural deficits [29]. In contrast to these studies on gray matter characteristics, research related to white matter parameters and sensorimotor control in individuals with NSLBP is lacking.

Therefore, the first objective of this study was to investigate potential differences in sensorimotor white matter microstructure in individuals with NSLBP compared to healthy controls. Second, we aimed to determine whether alterations in the sensorimotor white matter microstructure in individuals with NSLBP are associated with the capacity to weight proprioception for postural control. We hypothesized an impaired microstructural integrity of the sensorimotor white matter pathways in individuals with NSLBP compared to healthy controls, which is subsequently associated with an impaired proprioceptive weighting capacity.

## Methods

### Participants

Thirty-six individuals, aged between 20 and 50 years, voluntarily participated in this study. These participants were recruited by advertisement in the University Hospital Leuven, in sports clubs and in private practices. Individuals with a history of specific vestibular and/or balance problems, cardiovascular and/or neurological disorders, neck problems (Neck Disability Index [30] >6%) and previous major trauma and/or surgery of the spine or lower limbs were excluded. In addition, each individual had to meet the criteria for MRI related research. Forty-six patients with NSLBP were screened for participation. Eighteen individuals (12 women and 6 men) met all inclusion criteria and were included into the group with NSLBP. They had experienced at least six months of NSLBP and at least three episodes. Their score on the Oswestry Disability Index, version 2 (adapted Dutch version) (ODI-2) [31] was at least 12%. Eighteen individuals (13 women and 5 men) were included into the healthy control group. They did not have a history of NSLBP and reported a score of 0% on the ODI-2. A power-analysis based on previous studies [32], [33] revealed a sample size of 17 individuals per group as an adequate power to detect a clinically relevant difference in center of pressure (CoP) displacement during muscle vibration (0.80, 2-tailed, α = 0.05). All participants gave their written informed consent.

### Ethics Statement

The study conformed to the principles of the Declaration of Helsinki (1964), was approved by the local Ethics Committee of Biomedical Sciences, KU Leuven, Belgium (s53802) and registered at www.clinicaltrails.gov with identification number NCT01540617.

### Proprioceptive Weighting for Postural Control

Local muscle vibration was used to investigate the contribution of ankle muscle and back muscle proprioception to postural control. Local muscle vibration is a powerful stimulus of muscle spindle Ia afferents and evokes an illusion of muscle lengthening [34], [35]. Two muscle vibrators (Maxon motors, Switzerland) were applied bilaterally over the triceps surae muscles (‘ankle muscles’) and the lumbar paraspinal muscles (‘back muscles’). The proprioceptive stimulation was delivered by vibration at a high frequency and low amplitude (60 Hz, 0.5 mm) [34]. Prior to the measurements, individuals were presented with a few seconds of muscle vibration in order to avoid startle effects. When an individual used the proprioceptive signals from the vibrated muscle for postural control, a CoP displacement occurred. This mean CoP displacement, which is one of the most reliable indicators of the response to muscle vibration [36], was registered with a six-channel force plate (Bertec Corporation, OH, USA). Force plate data were sampled at 500 Hz using a Micro1401 data acquisition system and Spike2 software (Cambridge Electronic Design, UK). Mean values of the amount of CoP displacement were calculated using the following equation: CoP = Mx/Fz with Mx as the moment around the frontal axis and Fz as the vertical ground reaction force. The contribution of ankle and back proprioception, respectively, to postural control is represented in the amount of CoP displacement in response to muscle vibration [15], [16]. For example, a large CoP displacement in response to ankle muscle vibration demonstrated a large reliance on ankle muscle proprioception.

Throughout the experiment, the participants were asked to stand barefoot on a stable and unstable support surface (50 cm length×41 cm width×6 cm thickness, Airex balance pad elite). Their vision was occluded by means of non-transparent goggles and their arms were positioned relaxed along the body. They were instructed to try to maintain their balance during each trial (Table 1) and an investigator stood near the participant to prevent actual falls. A standardized feet position was used during the whole experiment with the heels placed 10 cm apart and a free forefoot position. After 20 seconds of upright standing, muscle vibration was applied bilaterally to the ankle or back muscles for 15 seconds. After vibration, participants were asked to preserve their position for 30 seconds. Between trials, one minute of rest was included and the participants were asked to move their ankles, knees, hips and lower back to reset muscle spindles. A similar setup was used in previous studies [16], [17], [37].

**Table 1:** Experimental set-up to evaluate proprioceptive weighting for postural control.

Trial	Description
**1**	Upright standing on **stable** support surface (20 s) – bilateral **ankle** muscle vibration (15 s)
**2**	Upright standing on **stable** support surface (20 s) – bilateral **back** muscle vibration (15 s)
**3**	Upright standing on **unstable** support surface (20 s) – bilateral **ankle** muscle vibration (15 s)
**4**	Upright standing on **unstable** support surface (20 s) – bilateral **back** muscle vibration (15 s)

### Diffusion MRI

Diffusion weighted single shot spin-echo echoplanar imaging (DTI SE-EPI) of the entire brain and brainstem was acquired with a Philips 3 Tesla Achieva scanner (Philips, Best, The Netherlands) and a standard head coil. Fifty-eight contiguous sagittal slices (slice thickness = 2.5 mm, voxel size = 2.5×2.5×2.5 mm^3^) with a data acquisition matrix of 96×96 mm^2^, a field of view of 200×240 mm^2^, a repetition time of 7600 ms and an echo time of 65 ms were obtained. Diffusion gradients were applied along 60 non-collinear directions, at a b-value of 1300 s/mm^2^. In addition, an average of five volumes without diffusion weighting (b = 0 s/mm^2^) was obtained. ExploreDTI 4.8.3 was used to analyze and process the data from the diffusion MRI acquisition [38]. The following multi-step procedure was used: (a) the quality of the diffusion MRI data was examined, (b) corrections for subject motion and eddy current induced geometrical distortions were performed, (c) a non-linear regression procedure was used to calculate the diffusion MRI parameters fractional anisotropy (FA) and mean diffusivity (MD). Besides FA and MD, the underlying eigenvalues radial diffusivity (RD) and axial diffusivity (AD) were calculated, (d) diffusion MRI data were co-registered to the Montreal Neurological Institute (MNI) space, and (e) the Johns Hopkins University (JHU) diffusion MRI-based white matter atlas was used to perform the correlation analysis. Diffusion MRI metrics were calculated by using an atlas-based approach [39]. Based on our anatomical hypothesis, we selected atlas-labels that are mainly involved in either sensory or motor processing or their combination to calculate the diffusion MRI metrics of these labels: the anterior limb of the internal capsule, the cerebral peduncle, the corticospinal tract, the inferior cerebellar peduncle, the medial lemniscus, the middle cerebellar peduncle, the posterior limb of internal capsule, the posterior thalamic radiation and the superior cerebellar peduncle.

### Statistical Analysis

Normality of the data was confirmed by the Shapiro-Wilks normality test (p>0.05). Group differences in baseline characteristics (p<0.05) (Table 2) and in white matter integrity were investigated by an unpaired t-test. CoP displacements were analyzed with a factorial between and within repeated measures ANOVA (p<0.05). Pearson correlations were calculated to investigate the association between proprioceptive weighting capacity for postural control and white matter metrics of the sensorimotor pathways. False discovery rate correction was applied to correct for multiple comparisons of group differences in white matter microstructure and correlation analysis. Based on the outliers labeling rule [40], no outliers were present in our study results. The statistical analysis was performed with SPSS 19.

**Table 2:** Characteristics of the participants.

	NSLBP group (n = 18)	Control group (n = 18)	p-value
**Age (yrs)**	33±8	31±8	(NS)
**Height (cm)**	173±6	169±6	(NS)
**Weight (kg)**	71±12	65±10	(NS)
**BMI (kg/m^2^)**	24±3	23±3	(NS)
**ODI-2**	22±8	0	N/A
**NSLBP (yrs)**	10±8	0	N/A
**NRS_back_ usual**	4.6±2.0	0	N/A
**NRS_back_ current**	2.1±2.0	0	N/A

Data are presented as mean ± standard deviation. NSLBP: non-specific low back pain; BMI: body mass index; ODI-2: Oswestry Disability Index, version 2 (adapted Dutch version); NRS_back_ usual: back pain score on the numerical rating scale (0–10) during the last month; NRS_back_ current: back pain score on the numerical rating scale (0–10) at the moment of testing; significance level (p<0.05); N/A: not applicable.

## Results

### Characteristics of the Participants

No significant differences in characteristics were found between individuals with NSLBP and healthy controls (p>0.05) (Table 2). Because no age difference between groups was observed (p>0.05) (Table 2), age was not included in the statistical analysis.

### Proprioceptive Weighting for Postural Control

No significant differences in proprioceptive weighting between individuals with NSLBP and healthy controls were observed (p>0.05).

### White Matter Microstructure

**Figure 1:**
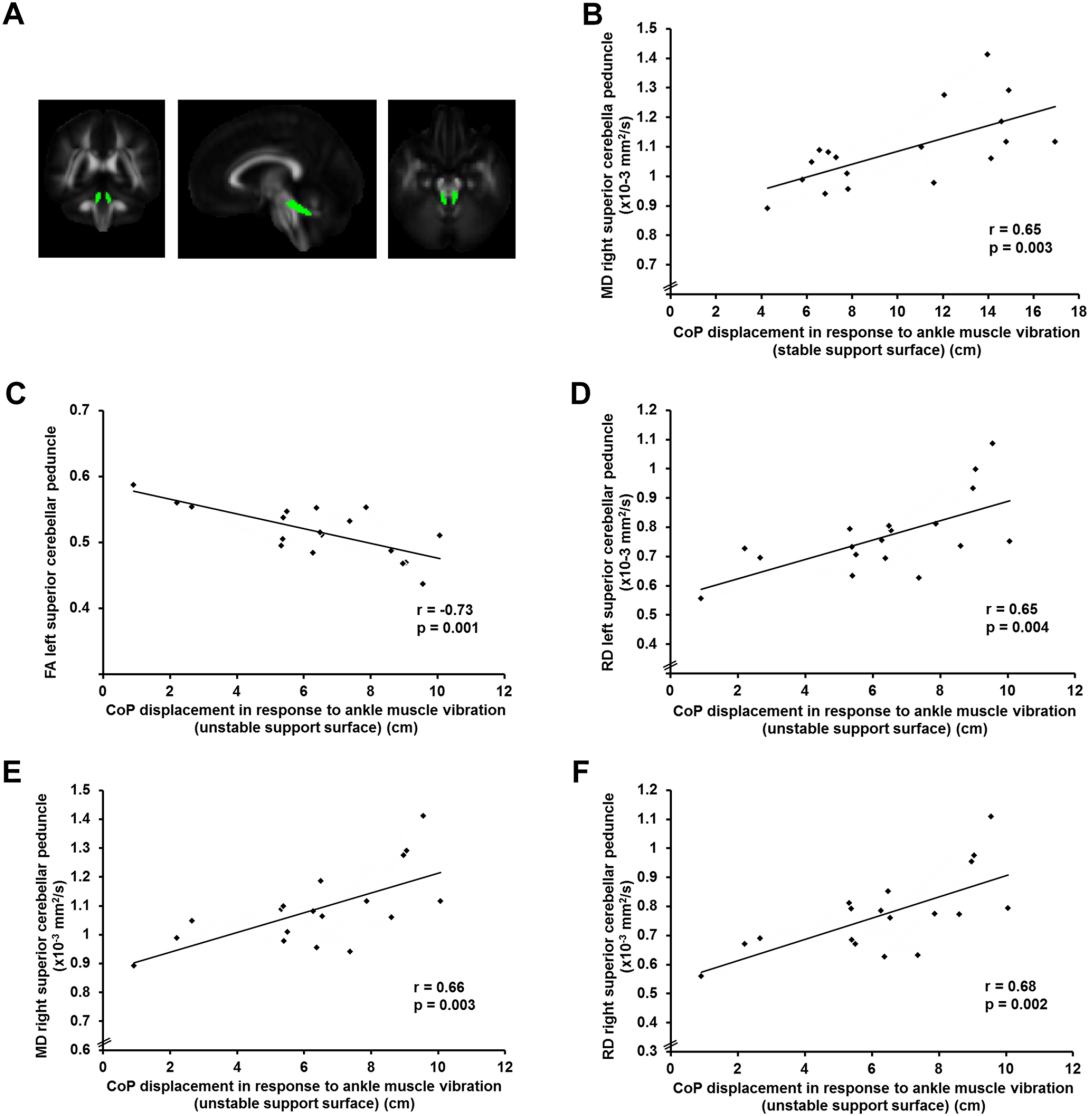
Association between white matter microstructure and proprioceptive weighting for postural control in individuals with NSLBP. **A.** Visualization of the right and left superior cerebellar peduncle. **B.** Scatter plot of the association between mean diffusivity (MD) of the right superior cerebellar peduncle and the center of pressure (CoP) displacement in response to ankle muscle vibration while standing on stable support surface in individuals with non-specific low back pain. **C.** Scatter plot of the association between fractional anisotropy (FA) of the left superior cerebellar peduncle and the center of pressure (CoP) displacement in response to ankle muscle vibration while standing on unstable support surface in individuals with non-specific low back pain. **D.** Scatter plot of the association between radial diffusivity (RD) of the left superior cerebellar peduncle and the center of pressure (CoP) displacement in response to ankle muscle vibration while standing on unstable support surface in individuals with non-specific low back pain. **E.** Scatter plot of the association between mean diffusivity (MD) of the right superior cerebellar peduncle and the center of pressure (CoP) displacement in response to ankle muscle vibration while standing on unstable support surface in individuals with non-specific low back pain. **F.** Scatter plot of the association between radial diffusivity (RD) of the right superior cerebellar peduncle and the center of pressure (CoP) displacement in response to ankle muscle vibration while standing on unstable support surface in individuals with non-specific low back pain.

No significant group differences in white matter microstructure did survive correction for multiple comparisons (p>0.003). However, a trend towards a reduced FA along the left superior cerebellar peduncle was found in the individuals with NSLBP compared to healthy controls (LBP: 0.517±0.039; HEA: 0.543±0.035; p = 0.039) (Figure 1A) (Table S1 and S2).

### Association between White Matter Microstructure and Proprioceptive Weighting in Individuals with NSLBP

MD of the right superior cerebellar peduncle was significantly correlated with the CoP displacement in response to ankle muscle vibration on stable support surface (r = 0.65, p = 0.003) (Figure 1B). On unstable support surface, the CoP displacement in response to ankle muscle vibration was significantly correlated with the FA and RD of the left superior cerebellar peduncle (FA: r = −0.73, p = 0.001; RD: r = 0.65, p = 0.004) and with the MD and RD of the right superior cerebellar peduncle (MD: r = 0.66, p = 0.003; RD: r = 0.68, p = 0.002) (Figure 1C–F). An impaired integrity of the white matter fibers passing through the superior cerebellar peduncle in individuals with NSLBP, which is represented by low FA and high RD and MD values, was associated with an increased reliance on ankle muscle proprioception. This is shown by larger CoP displacements in response to ankle muscle vibration. The CoP displacement in response to back muscle vibration in the individuals with NSLBP was not significantly correlated with the white matter metrics (p>0.003).

## Discussion

Individuals with NSLBP showed a trend towards a reduced FA along the left superior cerebellar peduncle compared to healthy controls, demonstrating a decreased microstructural integrity of this white matter pathway. Additionally, an impaired integrity of the white matter fibers passing through the superior cerebellar peduncle in individuals with NSLBP was associated with an increased reliance on ankle muscle proprioception. To the authors’ best knowledge, this is the first study showing an association between the microstructural integrity of a sensorimotor white matter pathway and the proprioceptive weighting capacity for postural control in individuals with NSLBP.

### Proprioceptive Weighting for Postural Control

Sensory weighting of proprioceptive input is required during postural control to efficiently adapt to the environmental conditions [8], [9]. On an unstable support surface, ankle muscle proprioception is less reliable due to the mismatch between the ankle joint angle change and postural sway [18]. A down-weighting of the less reliable ankle muscle signals is shown by a small CoP displacement in response to ankle muscle vibration [16]. This small CoP displacement indicates an efficient proprioceptive weighting capacity for this postural control [18]. Previous studies showed a decreased reliance on back muscle proprioception and an increased reliance on ankle muscle proprioception for postural control in individuals with NSLBP compared to healthy controls irrespective of the postural condition [16], [17]. In contrast, no significant differences in CoP displacement in response to muscle vibration between groups were found in the current study. Although, the current study sample consisted of older and more disabled individuals with higher levels of NSLBP compared to the younger and less disabled study samples in previous studies [16], [17]. More research is necessary to investigate whether these particular differences in characteristics are responsible for different responses to muscle vibration.

### White Matter Microstructure

Microstructural organization of the white matter pathways is a requirement for a smooth flow of information throughout the brain [41]. In this study, we found a trend towards a decreased integrity of the left superior cerebellar peduncle in individuals with NSLBP compared to healthy controls. This relatively small group difference is nonetheless of particular great interest, since we did not expect large structural brain lesions in this NSLBP sample (in contrast to e.g. patients with traumatic brain injuries [26] or multiple sclerosis [22]). The superior cerebellar peduncle is the main white matter pathway that conveys information from the cerebellum to other brain regions [42]. Therefore, we hypothesize that the impaired integrity of the superior cerebellar peduncle disturbs this information flow. This will result in an impaired integration of sensory signals, which occurs within specific cortical regions. In addition, degeneration of white matter pathways that connect the cerebellum to other brain regions, such as those passing through the superior cerebellar peduncle, might induce gray matter atrophy in some cerebellar regions that are crucial for balance control [22]. Therefore, the disrupted proprioceptive information inflow could be one of the causes of the reorganization in motor and sensory cortical areas in individuals with NSLBP [28], [29]. However, further studies are needed to examine this hypothesis.

### Association between White Matter Microstructure and Proprioceptive Weighting for Postural Control in Individuals with NSLBP

In the NSLBP sample, the microstructure of the superior cerebellar peduncle was significantly correlated with the CoP displacement in response to ankle muscle vibration. Specifically, a decreased integrity of the superior cerebellar peduncle in individuals with NSLBP was associated with an increased reliance on ankle muscle proprioceptive signals, even on unstable support surface and thus a weaker proprioceptive weighting capacity. This suggests that the superior cerebellar peduncle is an important sensorimotor pathway for proprioceptive signals during postural control in individuals with NSLBP. More specifically, it indicates that an impaired processing/transfer of proprioceptive information within/from the cerebellum impairs the weighting capacity for postural control in this sample.

Recently, the notion that the cerebellum is involved in sensory processing, and not only in motor coordination, has gained increasing attention [43]. The cerebellum plays an important role in sensory weighting, supporting the motor system with the best sensory information to adapt motor responses in a smooth and coordinated manner [43]. The theory of the superior cerebellar peduncle and the cerebellum as important contributors to the central processing of sensorimotor control is in accordance with previous studies. For example, associations between white matter deterioration of the cerebellum and/or superior cerebellar peduncle and impairments in sensorimotor control are found in individuals with traumatic brain injury [26], autism spectrum disorder [42] and multiple sclerosis [22]. In addition, abnormal postural sways and defective postural responses to external changes have been observed in individuals with cerebellar lesions [44]. Our finding that the microstructure of the superior cerebellar peduncle is an important contributor to sensory weighting for postural control in individuals with NSLBP, further supports this theory. In addition, these results emphasize the indirect link between the superior cerebellar peduncle and sensory data processing.

### Clinical Implications

Our findings contribute to a better understanding of the mechanisms and causes of NSLBP, which is essential to identify effective treatments [3]. The present findings suggest that impairments in central proprioceptive integration play a role in NSLBP, which implies the need for targeted proprioceptive rehabilitation in this population [45]. However, longitudinal studies are needed to investigate the effects of proprioceptive rehabilitation on central pathways in individuals with NSLBP, and to explore central alterations as a potential predictive factor in the maintenance and recurrence of NSLBP. Additionally, elucidating the mechanisms of sensorimotor processing for postural control may guide prevention and treatment strategies for individuals with impairments in postural control.

### Conclusion

This is the first study demonstrating a relationship between a reduced integrity of the superior cerebellar peduncle and a weak proprioceptive weighting capacity for postural control in individuals with NSLBP. These findings emphasize the importance of the superior cerebellar peduncle in proprioceptive weighting for postural control. Addressing these central mechanisms with targeted proprioceptive rehabilitation in individuals with NSLBP may proof fruitful. In addition, the results contribute to a better understanding of postural control, and open a window of further research on the central processing of proprioception for postural control.

## Supporting Information

Table S1 **Diffusion metrics (fractional anisotropy and mean diffusivity).** Diffusion metrics are presented as mean (M) and standard deviation (SD) for each region of interest (left (L) and right (R)) and for both groups. NSLBP: non-specific low back pain; Control: healthy individuals; p: p-value (bold value: p<0.05).
(PDF)

Table S2 **Diffusion metrics (axial diffusivity and radial diffusivity).** Diffusion metrics are presented as mean (M) and standard deviation (SD) for each region of interest (left (L) and right (R)) and for both groups. NSLBP: non-specific low back pain; Control: healthy individuals; p: p-value (bold value: p<0.05).
(PDF)
