# Supplementary material for: Microstructural Integrity of the Superior Cerebellar Peduncle Is Associated with an Impaired Proprioceptive Weighting Capacity in Individuals with Non-Specific Low Back Pain
Source: PLoS One. 2014 Jun 20;9(6):e100666. doi: 10.1371/journal.pone.0100666 (PMC4065054; doi:10.1371/journal.pone.0100666)
Supplement: Table S2 — Diffusion metrics (axial diffusivity and radial diffusivity). Diffusion metrics are presented as mean (M) and standard deviation (SD) for each region of interest (left (L) and right (R)) and for both groups. NSLBP: non-specific low back pain; Control: healthy individuals; p: p-value (bold value: p<0.05). (PDF) [file pone.0100666.s002.pdf]

Table S2. Diffusion metrics (axial diffusivity and radial diffusivity)

	Axial diffusivity (x10^{-3} mm^2/s)					Radial diffusivity (x10^{-3} mm^2/s)				
	NSLBP (n= 18)		Control (n= 18)			NSLBP (n= 18)		Control (n= 18)		
**Region of interest**	**M**	**SD**	**M**	**SD**	***p***	**M**	**SD**	**M**	**SD**	***p***
**Anterior limb internal capsule L**	1.069	0.040	1.060	0.029	0.428	0.461	0.027	0.462	0.024	0.932
**Anterior limb internal capsule R**	1.079	0.035	1.066	0.037	0.280	0.445	0.027	0.443	0.029	0.837
**Cerebral peduncle L**	1.389	0.054	1.380	0.039	0.541	0.443	0.030	0.461	0.033	0.101
**Cerebral peduncle R**	1.368	0.050	1.359	0.057	0.615	0.446	0.030	0.449	0.028	0.783
**Corticospinal tract L**	1.123	0.050	1.144	0.040	0.175	0.480	0.024	0.477	0.030	0.770
**Corticospinal tract R**	1.098	0.035	1.101	0.037	0.778	0.468	0.031	0.477	0.026	0.392
**Inferior cerebellar peduncle L**	1.135	0.047	1.130	0.041	0.761	0.504	0.035	0.512	0.032	0.474
**Inferior cerebellar peduncle R**	1.125	0.037	1.131	0.028	0.621	0.492	0.030	0.509	0.026	0.075
**Medial lemniscus L**	1.200	0.051	1.199	0.061	0.971	0.477	0.036	0.487	0.032	0.393
**Medial lemniscus R**	1.218	0.053	1.206	0.060	0.502	0.481	0.040	0.492	0.028	0.327
**Middle cerebellar peduncle**	1.142	0.039	1.162	0.055	0.225	0.527	0.032	0.543	0.033	0.156
**Posterior limb internal capsule L**	1.180	0.031	1.179	0.031	0.919	0.369	0.016	0.368	0.015	0.835
**Posterior limb internal capsule R**	1.174	0.039	1.175	0.028	0.946	0.356	0.020	0.363	0.019	0.324
**Posterior thalamic radiation L**	1.237	0.050	1.249	0.039	0.421	0.484	0.049	0.486	0.032	0.891
**Posterior thalamic radiation R**	1.233	0.054	1.231	0.040	0.917	0.473	0.026	0.469	0.027	0.695
**Superior cerebellar peduncle L**	1.699	0.144	1.667	0.117	0.468	0.768	0.130	0.698	0.101	0.081
**Superior cerebellar peduncle R**	1.721	0.135	1.696	0.124	0.565	0.773	0.137	0.731	0.103	0.308
